# Entropy of Neuronal Spike Patterns

**DOI:** 10.3390/e26110967

**Published:** 2024-11-11

**Authors:** Artur Luczak

**Affiliations:** Canadian Centre for Behavioural Neuroscience, University of Lethbridge, 4401, Lethbridge, AB T1K 3M4, Canada; luczak@uleth.ca

**Keywords:** information theory, neuronal analyses, spike train entropy

## Abstract

Neuronal spike patterns are the fundamental units of neural communication in the brain, which is still not fully understood. Entropy measures offer a quantitative framework to assess the variability and information content of these spike patterns. By quantifying the uncertainty and informational content of neuronal patterns, entropy measures provide insights into neural coding strategies, synaptic plasticity, network dynamics, and cognitive processes. Here, we review basic entropy metrics and then we provide examples of recent advancements in using entropy as a tool to improve our understanding of neuronal processing. It focuses especially on studies on critical dynamics in neural networks and the relation of entropy to predictive coding and cortical communication. We highlight the necessity of expanding entropy measures from single neurons to encompass multi-neuronal activity patterns, as cortical circuits communicate through coordinated spatiotemporal activity patterns, called neuronal packets. We discuss how the sequential and partially stereotypical nature of neuronal packets influences the entropy of cortical communication. Stereotypy reduces entropy by enhancing reliability and predictability in neural signaling, while variability within packets increases entropy, allowing for greater information capacity. This balance between stereotypy and variability supports both robustness and flexibility in cortical information processing. We also review challenges in applying entropy to analyze such spatiotemporal neuronal spike patterns, notably, the “curse of dimensionality” in estimating entropy for high-dimensional neuronal data. Finally, we discuss strategies to overcome these challenges, including dimensionality reduction techniques, advanced entropy estimators, sparse coding schemes, and the integration of machine learning approaches. Thus, this work summarizes the most recent developments on how entropy measures contribute to our understanding of principles underlying neural coding.

## 1. Introduction

Neurons communicate through electrical impulses known as action potentials or spikes, which serve as the fundamental units of neural signaling [1,2]. Sequences of these spikes over time, referred to as spike trains, encode information via the timing and frequency of spikes [3]. The precise temporal patterns within these spike trains are crucial for accurate information transmission across neural circuits [4,5]. Variations in these parameters significantly affect how sensory information is encoded and how motor commands are executed. For instance, the temporal structuring of spikes into bursts can enhance perceived sensory intensity, even when the overall spike rate remains constant [6]. Additionally, the reliability of spike timing plays a vital role in encoding complex stimuli, such as conspecific vocalizations, enabling neurons to transmit significant amounts of information beyond what is conveyed by spike count alone [7,8]. To better understand how neurons encode and transmit information, the concept of entropy from information theory has been applied to neural spike patterns [9,10]. Entropy quantifies the uncertainty or variability within a system, serving as a measure of information content [11]. In neuroscience, entropy is used to assess the variability of spike trains, providing insights into the amount of information that neuronal firing patterns can convey [12].

Understanding entropy in spike patterns is crucial for understanding the efficiency of neural coding mechanisms [2,13]. By quantifying the information content of neuronal responses, entropy offers a framework to analyze both the reliability and variability inherent in neuronal signaling [14]. High entropy in spike trains indicates a high degree of variability and potential information richness, while low entropy suggests more predictable patterns that could reflect either reliable signaling or reduced informational capacity [15].

However, focusing solely on individual spike trains may overlook the complex dynamics of cortical circuits, which communicate through coordinated patterns of activity involving thousands of neurons simultaneously. These high-dimensional spatiotemporal patterns are constrained by the underlying synaptic connectivity, resulting in only a subset of possible activity configurations being utilized for neural communication [16]. Consequently, information in the cortex appears to be conveyed as variations from common templates of activity rather than through entirely unrelated patterns for different stimuli [17]. This necessitates expanding entropy measures from single neurons to encompass multi-neuronal activity, a task that presents significant computational and theoretical challenges. Quantifying entropy in such complex neural data is essential for a deeper understanding of cortical information processing but requires novel approaches to handle the high dimensionality and interdependencies of neuronal networks. In the following section, we explore the concept of neuronal spike packets as a framework for understanding these coordinated activity patterns and their implications for neural coding.

### Neuronal Spike Packets

**Figure 1:**
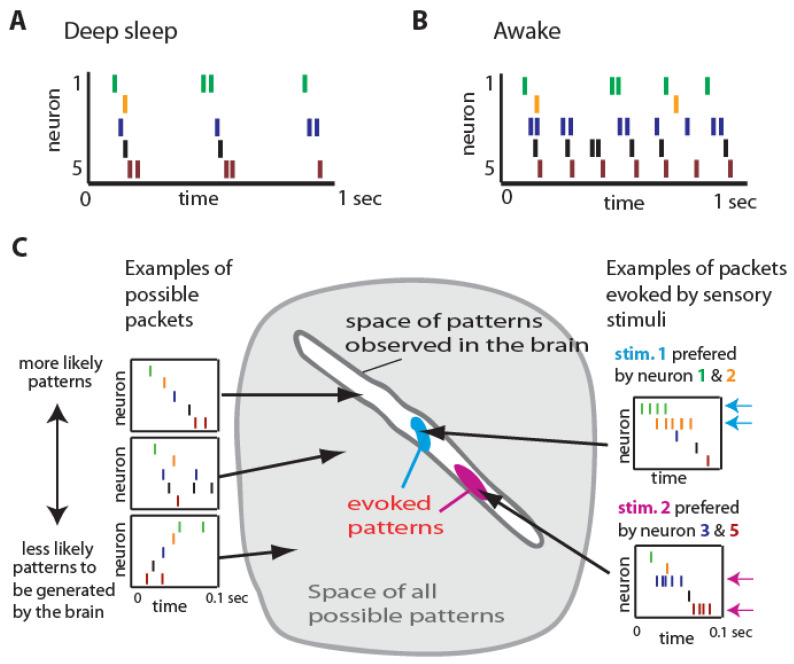
Cartoon illustration of neuronal activity packets. (**A**) Sequential activity patterns (called packets) during deep sleep where activity occurs sporadically. Within each packet, neurons fire with a stereotyped sequential pattern (each neuron marked with different color). (**B**) In an awake state, when more information is transmitted, packets occur right after each other, without long periods of silence, but temporal relationships between neurons are similar to those in the sleep state. (**C**) Consistency and variability in neuronal packets (geometrical interpretation). The gray area illustrates the space of all spiking patterns theoretically possible for a packet. The left-side panels show a cartoon of sample packets, each corresponding to a single point in gray space. The white area inside represents the space of packets experimentally observed in the brain. Packets evoked by different sensory stimuli occupy smaller subspaces (colored blobs). The right-side panels illustrate stimulus-evoked packets. The overall structure of evoked packets is similar, with differences in the firing rate and in the spike timing of neurons encoding information about different stimuli (figure modified from [18]).

An emerging concept in neuroscience is that cortical circuits communicate using coordinated patterns of neuronal activity, known as packets [16,18]. These neural packets are sequential bursts of spiking activity lasting approximately 50–200 milliseconds and are partially stereotypical in nature. As illustrated in Figure 1A, during deep sleep, these packets occur sporadically, with neurons firing in a stereotyped sequential pattern within each packet. In the awake state, packets occur more frequently, indicating increased information transmission while maintaining similar temporal relationships between neurons as in the sleep state (Figure 1B). This suggests that instead of continuous signaling, cortical communication occurs by sending discrete packets of activity, allowing for efficient and flexible information transmission across neural networks [19,20]. Neural packets consist of organized sequences of spikes across populations of neurons, containing both stereotypical structures and variable components that convey specific information [17]. The possible spiking patterns that a local neural circuit can produce are constrained by the neurons’ connectivity and intrinsic cellular properties. Consequently, certain activity patterns are more likely to emerge than others (Figure 1C, left panel). This idea can be visualized geometrically by representing each potential population spiking pattern as a single point within a space (Figure 1C, center panel). Experimentally, it was observed that spontaneous patterns occupy only a small subregion of this entire space of possible patterns [17]. Stimulus-evoked patterns are also limited by the same circuit constraints and form subspaces within the spontaneous pattern space. Each type of stimulus leads to variations in neuronal firing rates and, to a lesser extent, differences in spike timing, while the overall structure of the activity packet is preserved (Figure 1C, right panel) [18]. Thus, the stereotypical aspect refers to the consistent and repeatable patterns of spike activity observed across different packets, which likely reflect the underlying network architecture and synaptic connectivity. Variability arises from differences in spike timing and count between packets, enabling the encoding of diverse information within a consistent framework [16].

The partially stereotypical nature of these packets has significant implications for the entropy of cortical communication. Stereotypy reduces entropy by enhancing reliability and predictability in neural signaling, ensuring that essential information is consistently transmitted. Conversely, variability within packets increases the entropy of the system, reflecting greater information capacity and allowing for the transmission of specific details pertinent to sensory inputs or motor commands. This balance between stereotypy and variability enables cortical circuits to maintain both robustness and flexibility in information processing.

## 2. Quantifying Entropy in Spike Patterns

Understanding the informational content of neuronal spike patterns requires precise quantification methods from information theory. Several techniques have been adapted to neural data to measure entropy and mutual dependencies, providing insights into how neurons encode and transmit information.

***Shannon Entropy*:** Shannon entropy is a fundamental measure introduced by Claude Shannon in 1948 [9] to quantify the uncertainty or randomness in a set of possible outcomes. In neuroscience, Shannon entropy is used to measure the variability of spike trains by calculating the average information content per spike. To compute it, spike trains are often discretized into binary sequences over small time bins, indicating the presence (1) or absence (0) of a spike in each bin. The probability distribution of these binary patterns is then used in the entropy formula as follows:

$$H=-\sum_{i}p_{i}\;log_{2}p_{i}$$

where *p_i_* is the probability of occurrence of each distinct spike pattern *i*. A higher Shannon entropy indicates greater variability and potential information capacity in the neuron’s firing patterns. This method helps researchers assess the diversity of neural responses and the neuron’s ability to encode different stimuli [2,12].

***Entropy Rate*:** The entropy rate extends the concept of Shannon entropy to account for temporal correlations in spike trains. It measures the average uncertainty per unit of time, considering the dependencies between spikes at different times. Calculating the entropy rate involves estimating the joint probabilities of sequences of spikes over multiple time bins, which can capture patterns not evident when considering spikes independently. The entropy rate of a stochastic process is defined as follows:

$$H_{\mathrm{rate}}=\underset{n\to \infty }{\lim}\frac{1}{n}H\left(X_{1},X_{2},\ldots ,X_{n}\right)$$

where *H*(*X*_1_, *X*_2_,…, *X*_n_) is the joint entropy of the spike sequence over *n* time steps. This formulation reflects how the uncertainty grows with longer sequences of spikes and accounts for the dependencies across time, unlike the traditional Shannon entropy, which treats individual events independently. This method is particularly useful for neurons exhibiting burst firing or temporal coding strategies, as it reflects the information carried by the timing and patterns of spikes over time [12,21].

***Mutual Information*:** Mutual information measures the amount of information shared between two variables—in this case, the stimulus (S) and the neuronal response (R). It quantifies how much knowing the stimulus reduces the uncertainty about the response and vice versa. The mutual information is calculated using the joint probability distribution of stimuli and responses as follows:

$$I\left(S;R\right)=\sum_{s,r}p\left(s,r\right)\log_{2}\left(\frac{p\left(s,r\right)}{p\left(s\right)p\left(r\right)}\right)$$

where *p(s,r)* is the joint probability of stimulus *s* and response *r*, and *p(s)* and *p(r)* are their marginal probabilities. Mutual information captures both linear and nonlinear dependencies between stimuli and responses, making it a powerful tool for understanding neural coding efficiency. It can provide a direct measure of how much information about the stimulus is conveyed by the neuron’s spike patterns [11,13].

Mutual information can also be expressed in terms of **entropy** as follows:

$$I\left(S;R\right)=H\left(R\right)-H\left(R|S\right)$$

where *H*(*R*) is the entropy of the response, and *H*(*R*∣*S*) is the **conditional entropy** (see below). This expression shows that mutual information quantifies how much uncertainty about the neuronal response is reduced by knowing the stimulus. Thus, conditional entropy provides insight into how unpredictable the response remains even with knowledge of the stimulus, directly complementing mutual information by highlighting the variability that is not explained by the stimulus.

***Conditional Entropy*:** Conditional entropy can quantify the remaining uncertainty about the neuronal response given knowledge of the stimulus. It is defined as follows:

$$H\left(R|S\right)=-\sum_{s}p\left(s\right)\sum_{r}p\left(r|s\right)\log_{2}p\left(r|s\right)$$

where *p(r/s)* is the probability of response *r* given stimulus *s*. A lower conditional entropy indicates that the response is more predictable given the stimulus, suggesting higher reliability in encoding that stimulus feature [10]. In contrast, if conditional entropy is high, the neuron’s response varies significantly across instances of the same stimulus, indicating a noisier or less reliable encoding.

***Kullback–Leibler Divergence*:** While not an entropy measure per se, Kullback–Leibler (KL) divergence is often used to quantify the difference between two probability distributions. In neural data analysis, KL divergence can compare the observed spike train distribution to a reference model, such as a Poisson process. It is calculated as follows:

$$D_{KL}\left(P∥Q\right)=\sum_{i}p_{i}\log_{2}\left(\frac{p_{i}}{q_{i}}\right)$$

where *p_i_* is the observed probability distribution, and *q_i_* is the reference distribution. KL divergence indicates how much the neural firing patterns deviate from the reference, highlighting unique features of neuronal coding that may carry important information [22].

***Measuring entropy in dynamical systems:***

A dynamical system refers to a system where a set of variables evolves over time according to specific deterministic or probabilistic rules. These systems are widely studied across physics, biology, and neuroscience. In the analysis of such systems, entropy-based measures capture the degree of disorder and complexity in the evolving states. These measures are particularly useful for distinguishing between predictable and chaotic dynamics. Entropy measures like approximate entropy (ApEn) and sample entropy (SampEn) are prominent tools used to quantify the regularity and unpredictability of time series data [23,24]. ***Approximate entropy*** measures the likelihood that similar patterns in a time series will remain similar at the next point in the sequence, providing a robust estimate of system complexity in noisy environments. However, ApEn tends to overestimate the amount of regularity, leading to the development of SampEn, which refines this measure by excluding self-matching patterns and being less sensitive to data length and parameter settings [24,25,26]. These measures have been employed extensively in physiology and neuroscience, including applications, such as analyzing electroencephalogram (EEG) data, to assess changes in brain states, such as transitions between sleep stages or during cognitive load [27], where higher entropy values correspond to more complex and less predictable dynamics. For instance, using SampEn, researchers can distinguish between healthy and pathological brain states, where reduced entropy might signal a loss of complexity associated with diseases, such as schizophrenia or epilepsy [25]. These methods are particularly valuable in systems where traditional linear methods fail to capture non-stationary and nonlinear behaviors, underscoring the importance of entropy as a bridge between statistical descriptions and the dynamic evolution of complex systems [26].

## 3. Entropy as a Tool for Understanding Neuronal Processing

Entropy, as a measure of uncertainty or variability, has become an indispensable tool in unraveling the complexities of neuronal processing and neural coding strategies [11,13]. By quantifying the informational content of neuronal spike patterns, entropy enables researchers to dissect how neurons encode, transmit, and process information through their spiking activity [2]. Many studies have leveraged entropy-based methods to explore various facets of neural dynamics and neuronal information processing, as illustrated in the examples presented below.

Several studies have used entropy measures to investigate neural coding strategies. For example, Strong et al. [12] analyzed the entropy of spike trains in the visual system of flies, demonstrating how neurons maximize information transmission by balancing variability and precision. Similarly, DeWeese et al. [14] applied entropy calculations to auditory cortex neurons, revealing that neurons can transmit information with high temporal precision, thus enhancing the efficiency of neural codes. These applications highlight the relevance of entropy as a tool for understanding the balance between reliability and variability in neuronal communication, ultimately shedding light on how the brain processes and encodes information.

An important application of entropy in neuroscience is the investigation of critical dynamics in spiking neuron data. The concept of a critical regime refers to a balanced state between two extremes: one where neural activity spreads uncontrollably and another where activity quickly dies out. Operating at criticality allows the brain to optimize its capacity for information processing, enhancing both responsiveness and flexibility in neural communication [28]. Recent work by Lotfi et al. further illuminates this concept by identifying criticality signatures in cortical states using maximum entropy models in anesthetized rat brains. By segmenting data based on spiking variability, they observed that critical dynamics emerge within an intermediate range of variability, suggesting that cortical state shifts influence criticality and associated information processing. Their findings propose a universal dynamic, where the normalized distance to criticality collapses across cortical states, supporting a phase transition model within neural systems [29]. Serafim et al. [30] also used a maximum entropy approach based on firing rates to identify signatures of criticality in computational models and data from cortical neurons. Their results showed that neural networks exhibit behaviors indicative of phase transitions—abrupt changes in system dynamics—supporting the hypothesis that the brain may adjust its activity to operate near criticality for optimal functioning. Similarly, it has been shown that statistical complexity, measured using symbolic information theory, is maximized near this critical point in cortical spiking data [31]. By quantifying complexity across synchronized and desynchronized states, the findings revealed that complexity peaks at an intermediate state, aligning with the criticality hypothesis and suggesting an optimal balance between order and disorder for neural communication [31]. These studies highlight the utility of entropy-based models in capturing the complex dynamics of brain networks, showing that the brain’s tendency to operate near a critical point may be fundamental for efficient information transmission and adaptability in changing environments.

Entropy measures have also been instrumental in understanding the quality and limitations of neural models, particularly in describing large cortical populations. Olsen et al. [32] investigated the performance of pairwise maximum entropy (PME) models in capturing the spiking activity of large populations of neurons across various cortical areas. They found that while PME models perform well for small population sizes (N < 20), their performance diminishes for larger populations, indicating that these models may not adequately capture the higher-order interactions present in large neural networks. This limitation highlights the need for more sophisticated entropy-based models capable of accommodating the complexity of large-scale neuronal interactions [33,34].

Moreover, entropy-based methods have been applied to analyze pattern separation in neural circuits by leveraging concepts from information geometry. Information geometry is a mathematical framework that studies the relationships between probability distributions as points on a geometric surface, known as a manifold. In the context of neural activity, each point on this manifold represents a possible pattern of neuronal firing. Wang et al. [35] modeled pattern separation as the transformation of these patterns on a manifold, where small changes in input coordinates result in large geometric distances between output patterns. This reflects how even subtle differences in neural inputs can lead to distinct outputs, facilitating pattern separation. Using a two-neuron system, the authors demonstrated that existing similarity indices—commonly used to quantify how neural patterns differ—are highly sensitive to firing rate changes but fail to adequately capture differences in synchrony between neurons. This gap indicates the need for more robust entropy-based measures capable of capturing both firing rates and the temporal coordination of spikes, as both are critical for accurately quantifying neural information transmission [36,37].

Entropy also contributes to the development of computational models for interpreting neuronal data. Bardella et al. [38] introduced a mathematical framework based on lattice field theory to analyze neural systems, expanding the maximum entropy model to account for the time evolution of neural networks. Lattice field theory, originally developed in particle physics, represents complex systems as grids or lattices, where each point corresponds to a state of the system at a particular location and time. In neuroscience, this framework helps model neurons as discrete units interacting over time, similar to how physical particles interact on a lattice. Using this approach, Bardella et al. [38] captured both the spatial and temporal dynamics of neural networks, allowing for a more comprehensive analysis of collective neuronal behavior. Their methods enable researchers to interpret empirical observations from chronic neural interfaces—such as spike rasters—within a unified framework that links local neural interactions to broader network dynamics. This blending of concepts from particle physics and neuroscience offers new insights into brain processes, making it possible to predict and simulate complex neural activity with greater accuracy [33,34].

An example of information encoding in neuronal patterns was shown by Stasenko and Kazantsev [39], who investigated a mathematical model of a spiking neural network interacting with astrocytes. They found that astrocytic modulation prevented stimulation-induced hyperexcitation and non-periodic bursting activity, allowing the network to restore input images supplied during stimulation. This suggests that astrocytes play a role in homeostatic regulation of neuronal activity, with entropy measures capturing the effects of such modulation on the complexity and information content of neural signals [40,41].

An important consideration in applying entropy measures to neural data is ensuring that the temporal scales accurately capture underlying neural dynamics. Multiscale Entropy (MSE) is a method designed to assess how irregular a signal is across different time scales. Fine scales capture fast, small fluctuations, while coarse scales reflect slower, broader changes over time. This multiscale approach is particularly suitable for analyzing complex neural dynamics because neural systems can operate over a wide range of temporal and spatial scales. Traditional single-scale entropy measures may fail to capture this richness, making MSE a valuable tool for understanding the intricate patterns present in high-dimensional neural data. This method is intended to complement other neural measures, like signal variance and spectral power, by capturing nonlinear aspects of brain activity. Kosciessa et al. [42] critically evaluated MSE using simulated and real EEG data. Their study revealed that MSE’s results are often influenced by spectral power (the strength of different frequency components), leading to potential misinterpretations. Specifically, they found that coarse MSE scales, which should reflect slow dynamics, were biased by high-frequency components, while fine MSE scales, expected to capture fast dynamics, were strongly affected by low-frequency activity. This happens because MSE uses a similarity threshold to define patterns, which does not always align with the timescale of interest. This overlap complicates the interpretation of MSE—what looks like irregularity at one scale might actually reflect activity from a different frequency range. To address these issues, Kosciessa et al. proposed adjustments to reduce these biases, improving the precision of scale-specific entropy estimates. Their work underscores the importance of considering the interactions between entropy measures and the spectral properties of neural signals, advocating for best practices to ensure valid interpretations across time scales.

In addition, entropy measured at multiple scales has been utilized to study the impact of excitation–inhibition (E/I) balance on neural dynamics. Park et al. [43] examined how locally altered E/I balance affects neural connectivity, complexity, and information transmission. Their results showed that an increased E/I ratio strengthens excitatory connections but reduces the complexity of neural activity and decreases information transmission between neuron groups. This indicates that entropy can reflect changes in neural network dynamics resulting from imbalances in excitation and inhibition, which may have implications for understanding neuropsychiatric disorders characterized by altered E/I balance [44,45].

Entropy has also been instrumental in assessing the complexity of neural activity in relation to cognitive functions. Vivekanandhan et al. [46] analyzed spiking activity from the middle temporal area (MT) neurons and found that the Shannon entropy and conditional entropy were found to be capable of capturing the working memory content. This suggests that complexity measures derived from entropy can capture the modulation of neural activity associated with cognitive processes, such as working memory, offering potential biomarkers for cognitive states [47,48].

Entropy is also used in image processing, where higher entropy values denote greater shape irregularity [49]. Thus, entropy measures can also be applied to images of neurons, providing valuable insights into their complexity and spatial distribution. A neuron with a highly branched, irregular dendritic structure would exhibit higher entropy, while a simpler, more uniform structure would have lower entropy. Analyses of neuronal patterns not only can provide a quantitative measure of dendritic complexity but also can help to study environmental factors contributing to the diversity of neuronal morphologies observed in the brain [50,51,52].

*Predictive coding*: The brain is increasingly conceptualized as a prediction machine that continuously generates and updates internal models to anticipate sensory inputs and minimize prediction errors [53,54,55,56,57]. Predictive coding frameworks posit that the brain actively infers the causes of its sensations by reducing the discrepancy between expected and actual sensory input [58,59]. However, this process is not solely about minimizing entropy or uncertainty in neural representations. Instead, it involves the broader principle of minimizing variational free energy, which balances the trade-off between the accuracy of sensory predictions and the complexity of the internal models generating them [53]. Variational free energy comprises two key components: prediction error (reflecting accuracy) and complexity (reflecting the simplicity of the model). By minimizing free energy, the brain strives to optimize this balance, ensuring that its models are precise enough to explain sensory inputs without becoming unnecessarily complex [53,60]. Entropy reduction is part of this optimization but represents just one facet of the overarching goal of minimizing prediction error [61].

## 4. Challenges and Future Directions

One of the significant challenges in analyzing the entropy of neuronal spike patterns, particularly within neuronal packets, is the “**curse of dimensionality**”. Neuronal packets can involve coordinated activity across vast populations of neurons—potentially millions—over time scales of hundreds of milliseconds [16,62]. To capture the intricate spatiotemporal dynamics within these packets, researchers often divide the data into fine temporal bins ranging from 1 to 5 milliseconds [17]. This granular approach results in a high-dimensional data space, where each neuron’s activity in each time bin represents a separate dimension. Consequently, estimating entropy in such a high-dimensional space becomes increasingly complex and less reliable due to the exponential growth in computational and data requirements [63].

The curse of dimensionality poses several problems for entropy estimation in neuronal data. Firstly, as dimensionality increases, the volume of the data space expands exponentially, causing data points (spike patterns) to become sparser relative to the space they occupy [64]. This sparsity makes it challenging to obtain accurate probability distributions necessary for entropy calculations since traditional estimation methods require an impractically large amount of data to sample the space adequately [65,66]. For instance, in a system with just 100 neurons binned over 100 time points, the number of possible spike patterns exceeds the number of atoms in the observable universe, rendering exhaustive sampling infeasible [67].

Moreover, high dimensionality affects the reliability of entropy measures due to increased variance and bias in estimators. Estimators such as the plug-in method or histogram-based approaches become less effective because they suffer from bias when data are insufficient to populate the high-dimensional bins [12,68]. Nearest neighbor estimators, while more data efficient, also face challenges as distances between points become less meaningful in high dimensions [69]. These issues can lead to inaccurate assessments of the informational content of neuronal spike patterns, hindering our understanding of neural coding mechanisms.

To address these challenges, several strategies have been proposed to mitigate the curse of dimensionality in entropy estimation. One approach is to employ dimensionality reduction techniques that project high-dimensional data onto a lower-dimensional representation while preserving essential features of the data [70,71]. Methods such as principal component analysis (PCA) or more advanced nonlinear techniques, like t-distributed stochastic neighbor embedding (t-SNE), can reduce the effective dimensionality, making entropy estimation more tractable [72]. For example, in studies of neuronal patterns, applying methods like PCA can identify principal components that capture the majority of variance in neural activity, thereby simplifying the entropy calculation without significant loss of information [73].

Another strategy involves developing advanced entropy estimators that are more robust to high dimensionality. Adaptive methods, such as the Bayesian entropy estimator or the k-nearest neighbor estimator, adjust their parameters based on the data distribution, providing more accurate estimates with limited data samples [74,75]. These estimators can better handle the sparsity of data in high-dimensional spaces by effectively utilizing the available information. For instance, the Kozachenko–Leonenko estimator has been successfully applied to estimate the entropy of high-dimensional neural data with improved accuracy [75]. To illustrate, consider neuronal spike train data, which are often high-dimensional and sparse because neurons often fire infrequently. Traditional entropy estimators, like histogram-based methods, struggle in this context because they require dividing the data into bins; with sparse data, many bins remain empty, leading to unreliable entropy estimates. In contrast, the Kozachenko–Leonenko estimator bypasses the need for binning by calculating the distances between each data point and its nearest neighbors. This adaptability enables more precise entropy estimates, even when dealing with sparse and high-dimensional neural datasets, making it particularly suitable for analyzing complex neural dynamics.

Exploring sparse coding schemes offers another avenue to mitigate dimensionality challenges. The brain may utilize sparse representations, where only a small subset of neurons is active at any given time, reducing the dimensionality of the active neural space [76,77]. By modeling neural data under the assumption of sparsity, entropy estimations become more manageable, and the relevant informational content can be extracted more efficiently. This approach aligns with evidence suggesting that neuronal packets may operate under principles of sparse and efficient coding to optimize information transmission [78].

Looking forward, integrating machine learning techniques holds promise for addressing high-dimensional entropy estimation. Deep learning models, particularly those designed for high-dimensional data, such as convolutional neural networks (CNNs) or recurrent neural networks (RNNs), can learn compact representations of neural data [79,80]. By training these models on spike patterns, one can extract latent variables that capture the essential dynamics of neuronal packets, effectively reducing dimensionality. Furthermore, generative models, like autoencoders or generative adversarial networks (GANs), can model the underlying probability distributions of neural data, facilitating more accurate entropy estimation [81,82,83].

Another future direction involves improving experimental designs to collect data that are more amenable to entropy analysis. Advances in neural recording technologies, such as high-density electrode arrays and optical imaging techniques, enable simultaneous recording from larger populations of neurons with high temporal resolution [67,84,85,86,87]. Carefully designed experiments that selectively target relevant neural populations or time periods can reduce the dimensionality of the data while preserving critical information about neuronal packets.

Implementing entropy-based analyses in neural data research presents several practical challenges, particularly concerning the limitations inherent in fMRI and EEG modalities. For instance, fMRI data, characterized by low temporal resolution and susceptibility to motion artifacts, can complicate the accurate estimation of entropy measures. To address these issues, researchers have developed novel windowing approaches that select and concatenate low-motion segments of fMRI data, thereby reducing the impact of motion on sample entropy estimates [88].

## 5. Conclusions

Entropy serves as a powerful quantitative framework for understanding neuronal processing across multiple levels, from single neurons to large-scale networks. By quantifying the variability and information content of neuronal spike patterns and packets, entropy-based methods provide critical insights into neural coding strategies, network dynamics, synaptic plasticity, and cognitive functions. These applications underscore the importance of entropy in advancing our understanding of brain function and highlight the potential for future research to further exploit entropy in neuroscience. While the curse of dimensionality presents a significant hurdle in estimating the entropy of neuronal spike patterns, particularly within neuronal packets, it also opens avenues for methodological innovation. By adopting dimensionality reduction techniques, developing advanced entropy estimators, leveraging models that exploit neural data structure, and integrating machine learning approaches, researchers can mitigate these challenges. Addressing the high dimensionality inherent in neuronal patterns is essential for advancing our understanding of neural coding and information processing in the brain.

Future research could explore the application of entropy measures to study the temporal evolution of neuronal packets, providing insights into how information is dynamically processed and integrated over time. Additionally, investigating the role of entropy in understanding the impact of neurological disorders on neuronal communication could offer novel perspectives on disease mechanisms and potential therapeutic targets. Continued efforts in this direction will enhance our ability to decipher the complex language of neuronal communication and unravel the fundamental principles underlying brain function.
